# A Curious Congenital Deformity

**Published:** 1894-05

**Authors:** Ella M. Tuttle

**Affiliations:** Philadelphia, Pa., Resident Physician Hospital of Women’s Homœopathic Association


### A CURIOUS CONGENITAL DEFORMITY.

ELLA M. TUTTLE, M. D., PHILADELPHIA, PA.,
Resident Physician Hospital of Women's Homœopathic Association.

Mrs. C——, multipara, aged twenty, was delivered at the Women's Homœopathic Hospital, Maternity department, on the 22d of February, of a male child weighing eight pounds. She had a short, uneventful labor, and no unusual force was used or needed in delivering. When expelled from the vagina the left leg of the child was straightened across the abdomen and chest, and on examination the knee was found to be dislocated. The bones of the knee-joint were normal in their development, and the folds of the skin over the patella were filled with *vernix caseosus*, showing that the dislocation had existed for some time.

Dr. Walter Strong, our house surgeon, was called in consultation, and the dislocation was easily reduced, and the leg confined in a flexed position by a posterior splint and bandage. A single dose of *Acon.*[30] was given. February 24th bandage alone was applied, flexion being secured by a strap passed from the heel to the hip. There was a very little swelling of the knee February 26th, but this had disappeared the next day.

From March 5th to March 13th a flexible splint was applied to the outer side of the leg, then all bandages were discontinued, as there now seemed no tendency to dislocation. The child remained under my observation till March 22d, when it was discharged with its mother. At this time the knee seemed perfectly well and the child flexed and extended both legs synchronously. The only cause that we could assign for the dislocation was a fall that the mother had in December, and from which she had suffered some pain and soreness for several days. Aside from this she was exceptionally well all through her pregnancy.

By permission of committee.

